# Preliminary Examination of the Effects of Focused Ultrasound on Living Skin and Temperature at the Skin–Transducer Interface

**DOI:** 10.3390/bioengineering11111126

**Published:** 2024-11-08

**Authors:** Andrew A. E. D. Bishay, Andrew J. Swenson, Norman M. Spivak, Samantha Schafer, Brendan P. Bych, Spencer D. Gilles, Christopher Dorobczynski, Alexander S. Korb, Mark E. Schafer, Taylor P. Kuhn, Martin M. Monti, Alexander Bystritsky

**Affiliations:** 1 Department of Psychiatry and Biobehavioral Sciences, UCLA, 760 Westwood Plaza, Suite C8-519, Los Angeles, CA 90095, USA; andrewbishay@ucla.edu (A.A.E.D.B.);; 2 Weill Cornell Medical College, Cornell University, 525 East 68th Street, New York, NY 10065, USA; 3 Department of Neurosurgery, University of California Los Angeles, 760 Westwood Plaza, Suite C8-519, Los Angeles, CA 90095, USA; 4 School of Biomedical Engineering, Science and Health Systems, Drexel University, 3141 Chestnut St, Philadelphia, PA 19104, USA; 5 Department of Neurosurgery, School of Medicine, Tulane University, New Orleans, LA 70118, USA; 6 Kaiser Permanente Oakland Medical Center, Oakland, CA 94611, USA; 7 Department of Environmental Studies, University of Southern California, Los Angeles, CA 90007, USA; 8 Department of Psychology, University of California Los Angeles, 760 Westwood Plaza, Suite C8-519, Los Angeles, CA 90095, USA

**Keywords:** ultrasound, temperature, heating, skin

## Abstract

Transcranial Focused Ultrasound Stimulation (tFUS) is a new, rapidly growing field related to the study and treatment of brain circuits. Establishing safety cutoffs for focused ultrasound is crucial for non-ablative neurological ultrasound experiments. In addition to potential focal heating, there is concern about temperature elevation at the skin surface. Much work has been performed at or near the FDA guideline of I_SPTA.3_ = 720 mW/cm^2^, which technically only applies to diagnostic, not therapeutic, ultrasound. Furthermore, evidence of brain tissue damage on histology in the focal region has been shown not to occur until I_SPTA.3_ > 14 W/cm^2^. Therefore, this study was conducted across a range of intensities between these two values, evaluating both subjective and objective side effects. Subjective side effects encompassed any discomfort experienced during and after focused ultrasound stimulation, while objective side effects included clinical findings of skin irritation, such as erythema, edema, or burns. This study also examined how the skin temperature at the skin–transducer interface would change in order to assess whether there would be significant heating. The subjects did not experience any unpleasant sensation at the point of stimulation, including heat or pain, and no objective findings of skin irritation were observed following stimulation and the removal of the transducer. In addition, there was no intensity-dependent effect on temperature, and the maximal rise in temperature was 1.45 °C, suggesting that these parameters do not result in the heating of the skin at the interface in such a way that poses a risk to subjects when operating at or below the intensities tested in this experiment.

## 1. Introduction

Focused ultrasound stimulation (FUS) is a promising treatment modality that can potentially target almost any region in the brain and is especially suitable for deep, subcortical structures. Furthermore, because ultrasound waves can be focused in three dimensions, they have high spatial specificity and a minimal effect on other areas. FUS allows for the noninvasive delivery of acoustic energy to a well-localized and circumscribed brain region of a few millimeters in diameter. Previous studies have demonstrated the transcranial application of FUS to deep brain areas using either a single-element transducer or an array of multiple ultrasound transducers [1,2].

Yoo and colleagues showed that ultrasound could selectively suppress regional neural activity in the rabbit brain [3]. Importantly, these effects were reversible and did not result in significant temperature change. While high-intensity focused ultrasound (HIFU) is used for ablative treatments, the energies used in FUS for neuromodulation are an order of magnitude lower than those used in HIFU. In addition, while HIFU typically is administered using continuous-wave (CW) ultrasound, low-intensity FUS (LIFU) is administered in short pulses, further reducing the total energy deposition. Many groups have shown that the administration of LIFU causes a significant but reversible neuromodulatory effect while avoiding tissue damage [4,5,6].

In a safety study of refractory temporal lobe epilepsy in patients undergoing epilepsy surgery, the histological examination of temporal lobe brain tissue sonicated at derated spatial-peak, temporal-average intensities (I_SPTA.3_) of 720, 1440, 2880, and 5760 mW/cm^2^ yielded normal, unremarkable findings [7]. Furthermore, ex vivo studies of freshly resected human brain tissue have shown that histological abnormalities—such as apoptosis, necrosis, cavitation, and spongiosis—do not appear until intensity levels exceed I_SPTA.3_ = 14 W/cm^2^ [8]. Therefore, it was of interest to investigate whether increasing focal intensities would result in damage to living skin and elevated skin temperatures.

A potential safety limitation of transcranial ultrasound stimulation is that a significant portion of the energy is reflected by the skull and absorbed by the skin. Therefore, before performing in vivo experiments on human brains at these higher intensity levels, we designed the present study to determine the potential for burns if the absorbed energy was sufficiently large, and the potential of ultrasound exposure at these levels to cause significant tissue heating.

This study was conducted to test a worst-case scenario. Many brain ultrasound studies take advantage of a thin, flat region of the skull, known as the temporal window [8]. The scapula affords a large flat reflecting area, lying parallel to the skin surface. This geometry enhances the potential for energy to reflect within the skin overlying the scapula. Further, the skin overlying the scapula is relatively thin, thus offering a small thermal mass, creating optimal conditions to test for possible heating. Finally, the scapula is almost double the temporal window’s thickness and will absorb nearly all incident ultrasound waves [9], providing another potential heating source for the overlying skin. The potential heat deposition is primarily affected by the thickness of skin and tissue overlying the bone, the vascularity of that tissue, and the absorption of ultrasound energy in the bone. The heat sources are the transducer itself, and the conversion of ultrasound energy into heat by absorption in the overlying tissue and in the bone. These factors do not change depending on whether the ultrasound source is in the temporal window or elsewhere on the skull. Therefore, the scapula was chosen because it can provide a surface that can be in complete contact with the transducer, such as in the temporal window.

It is well recognized that proper acoustic coupling is required to ensure effective treatment and to avoid adverse events. This is true for general ultrasound diathermy as well as lithotripsy devices. The worst-case scenario depends upon several factors, specifically the spatial-peak temporal-average intensity (I_SPTA_) desired at the focal region, the focal gain or F# of the system, and the operating frequency. The focal gain is involved because the higher the focal gain, the lower the energy density at the transducer face relative to the focal intensity, I_SPTA_. Obviously, the maximum I_SPTA_ for the case of neuromodulation is significantly lower than that of HIFU, which is designed to heat tissue to the point of necrosis. Using the focal gain of this transducer, we can work backward to determine the total power required, and then use the Thermal Index Cranial (TIC) to estimate the resultant heating at the skull surface. The resulting maximum value is 5.11. However, it must be noted that the TIC calculation assumes that all the ultrasound energy is absorbed by the bone and that an exposure time is sufficient to achieve the steady-state condition, which is clearly not the case. At 650 kHz with a 60 mm diameter transducer, at least 50% of the ultrasound energy passes through the skull, which would reduce the maximum TIC by a factor of two [10]. Therefore, the worst case for the transducer/frequency combination would be a revised TIC of approximately 2.55.

For the highest exposure level, the derated peak rarefactional pressure estimated within the skull, factoring in losses caused by transiting the skull, would be 1.4 MPa, with an MI of 1.7. These values are well below those that would be expected to create cavitation within tissue that does not contain microbubbles (contrast agents). These levels are also below the comparable FDA guideline limit for diagnostic ultrasound of MI = 1.9. That being said, exposures with higher focal peak rarefactional pressures may lead to unwanted bioeffects due to cavitation action. For instance, histotripsy, with peak rarefactional pressures above 10 MPa, causes the cavitation-induced liquefaction of tissue [11]. Such levels should clearly be avoided in neuromodulation therapies.

As such, this study aimed to explore the safety of the BrainSonix BX Pulsar 1002 Focused Ultrasonic Pulsation Device at a stimulation of I_SPTA.3_ ≤ 14 W/cm^2^ by examining the subjective and objective side effects of administering FUS to the infraspinous fossa of the scapula and monitoring changes in the surface skin temperature, as measured by thermocouple probes placed on the skin beneath the transducer.

## 2. Materials and Methods

### 2.1. FUS Device

**Figure 1:**
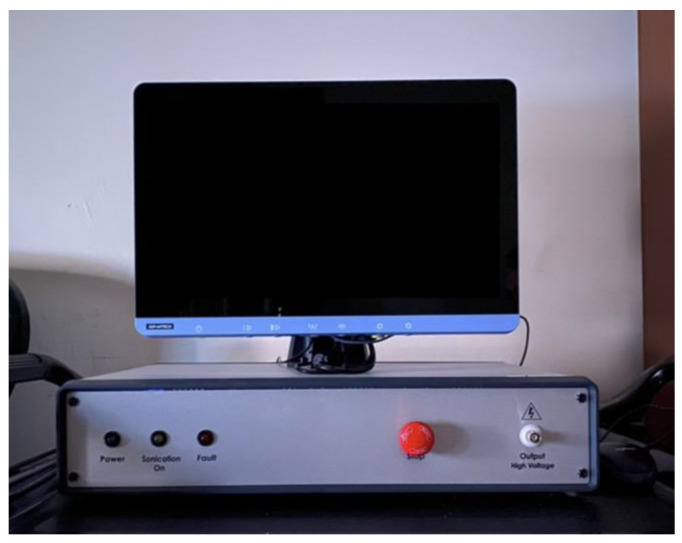
Brainsonix BX Pulsar 1002 ultrasound console.

**Figure 2:**
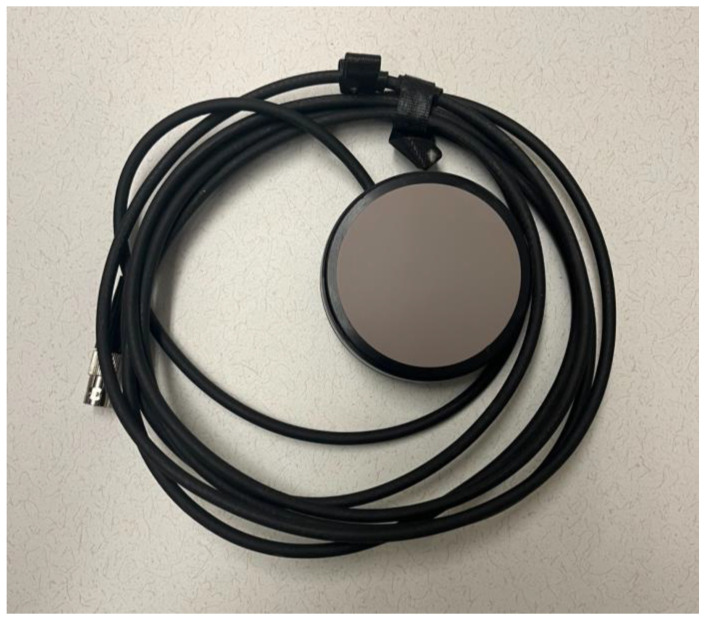
Brainsonix BX Pulsar 1002 FUS transducer.

BrainSonix (Sherman Oaks, CA, USA) designed the BX Pulsar 1002 (Figure 1) to deliver FUS to the human brain [2]. The BX Pulsar 1002 consists of two main elements, the transducer and the ultrasound console, which integrates various components such as a function generator, power meter, amplifier, computer, and isolation transformer. The ultrasound console controls the pulse shape, tone burst duration, and pulse repetition frequency of the signal transmitted to the transducer. It also contains specially designed software to ensure safety by monitoring and controlling the output power. Acertara Acoustic Laboratories (Longmont, CO, USA) assembled the ultrasound console in accordance with documented procedures. This study utilized a BrainSonix transducer with a 61 mm diameter and a nominal 55 mm focal depth (Figure 2). The transducer’s focal zone (region in which the acoustic pressure was greater than half of the maximum, or −6 dB) extended from 44 to 68 mm in depth and was 3.8 mm in width (circular cross-section).

### 2.2. Thermistor

We made temperature measurements using three NK272C1B1 thermistors (Amphenol Thermometrics, St. Marys, PA, USA) with a 3 mm × 4 mm exposed tip and a nominal resistance of 2700 Ohms at room temperature and a mass of 180 mg; their “B value” in the range of 25° to 85 °C was 3977. To measure temperature, we placed the thermocouple in series with a 2700 Ohm resistor and applied a fixed voltage reference of 3.3 volts. The voltage across the thermocouple was measured using a Feather M0 express microcomputer (Adafruit, New York City, NY, USA) using its built-in 12-bit analog-to-digital converter, resulting in a voltage resolution of approximately 800 µV. To calibrate each thermistor, we cycled their temperatures from 20 °C to 60 °C while measuring the absolute temperature with an LM34Z temperature-to-voltage converter (Texas Instruments, Dallas, TX, USA). For each temperature measurement, we collected 11 samples at a rate of 1 kHz and averaged the results.

### 2.3. Participants

We conducted the study in accordance with the ISO 14155-1:2003 [12], Clinical investigation of medical devices for human subjects—Part 1: General requirements, the Good Clinical Practice (GCP) requirements according to the ICH Guidelines and applicable national laws and regulations, and the ethical principles that have their origin in the Declaration of Helsinki. In addition, the University of California, Los Angeles (UCLA), Institutional Review Board approved all study materials and procedures (UCLA IRB: 18-001067), and all recruited volunteers signed informed consent forms before participating. Eleven subjects participated in the study.

### 2.4. Procedure

We conducted the study using UCLA as a single site, with one visit involving an initial evaluation and FUS administration. The entire visit took approximately one hour. We recruited subjects in good general health, 18 years or older, without restrictions on race or gender. While there are no known risks regarding the delivery of ultrasound at the proposed power levels, we nonetheless excluded participants with (i) cognitive or psychiatric disorders that may limit their ability to give informed consent or render them unable to cooperate with the testing; (ii) severe cardiac disease or increased intracranial pressure, using a transcutaneous electrical nerve stimulation (TENS) unit, (iii) implanted medical devices; (iv) a history of a seizure disorder; (v) a history of substance abuse; or (vi) who were pregnant at the time of enrollment.

The present experiment delivered ultrasound to the research subjects’ right scapula (shoulder blade) at the infraspinous fossa. We asked participants to remove their shirts and to lie in a supine (face up) position on a bed. We used three thermocouple probes placed on the skin: one under the active transducer (right shoulder), one under a sham transducer (left shoulder), and one reference probe, which was placed on the spine approximately in the midpoint between the two shoulder locations. The sham transducer was of the same design and model as the active transducer but was not energized. It was used to determine the warming effects of occluding the skin surface with the gel and transducer, without ultrasound excitation. We applied ultrasound gel in sufficient quantity to facilitate the uninterrupted transmission of the ultrasonic signal through the transducer-thermocouple–skin interface. At the time of sonication, we did not tell the subjects which transducer delivered ultrasound pulses and which was used as a sham location.

After we fitted the participants with the transducers, they underwent FUS stimulation at five levels of intensity (I_SPTA.3_ = 6 W/cm^2^, 8 W/cm^2^, 10 W/cm^2^, 12 W/cm^2^, and 14 W/cm^2^), using the following parameters: 0.5 ms Tone Burst Duration, 100 Hz pulse repetition frequency, 5% duty cycle, and 1 min sonication duration. These are the parameters most frequently used by groups for neuromodulation experiments.

During each session, we delivered one sonication at each of the five intensity levels listed above (for a total of five sonications); each sonication lasted 1 min, for a total of 5 min of sonication (Table 1). There was about 2 min in between sonications. However, with the specified duty cycle of 5%, FUS was transmitted only during 5% of the total 5 min sonication period. This corresponds to a total active ultrasound exposure time of 15 s. In addition, the order in which the five different intensity levels were delivered was randomized so as to not bias the results, i.e., a potential cumulative heating effect could make the later sonications appear to produce more heating. During and after the sonication, the subjects were asked every few minutes about the sensation of heating or pain or any other unpleasant sensation under the transducer.

## 3. Results

Across all 11 subjects, there were no subjective side effects reported. This included periods in which the transducer was placed, before the sonication protocol had begun, during the sonication protocol, and the follow-up period after the protocol had been completed.

There were no objective side effects observed as a result of higher-intensity FUS stimulation. Upon visual assessment of the skin underlying the electrodes and transducers, there were no signs of erythema, swelling, irritation, or burns.

We designed the sonication protocol with a sham transducer on the opposing shoulder. This created a baseline against which temperature changes in the active sonication site could be compared. Notably, the subjects failed to accurately identify the shoulder receiving active stimulation. Temperature was also recorded at a third location midway between the shoulders on the spine, which also provided another baseline against which temperature change could be compared. Skin temperature was tracked during the course of the sonication protocol. One minute of continuous FUS stimulation resulted in a peak temperature change of 1.45 degrees C, observed at any intensity of I_SPTA.3_ = 12 W/cm^2^. Notably, this was below the safety cutoff of 5 degrees C established a priori for the study. It is important to note that this is a hard stop for cutting off any further stimulation. As we proposed working with increasing intensities, the FDA cutoff of 2 degrees was impractical, because we thought it would produce too many false positives. Post hoc, a 2-degree cutoff would have been sufficient since the peak change was 1.45 °C.

**Figure 3:**
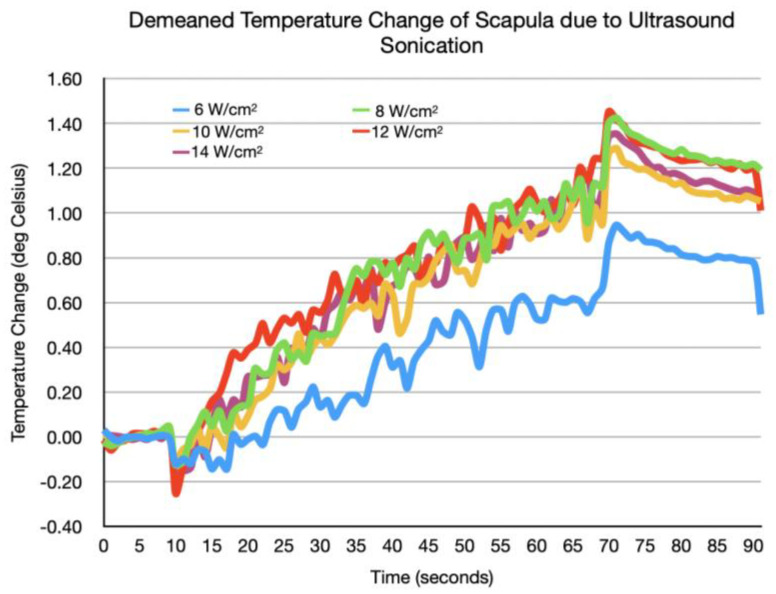
Change in temperature following ultrasound stimulation at different intensity levels.

The peak change in temperature across other tested intensities was also below this cutoff and is recorded in Table 2. Figure 3 records the demeaned temperature changes recorded from the 10 s before beginning active stimulation to the end of the 1 min stimulation period, across the tested stimulation intensities. The peak temperature change did not appear to have a dose-dependence effect on the ultrasound intensity. At all intensity levels, a peak temperature change of 0.92–1.45 °C was seen. The temperature returned to baseline during every inter-sonication interval.

The marginal mean temperature changes are reported in Table 3. The mean increase at 6 W/cm^2^ was significantly lower than the increases at 8–14 W/cm^2^. However, there was no significant difference in the marginal mean temperature increase between intensities 8 W/cm^2^ and higher.

## 4. Discussion

The peak temperature change in the scapula area under the transducer during LIFU administration at intensities between 6 W/cm^2^ and 12 W/cm^2^ remained under 2 °C. This does not exceed the FDA-established threshold for diagnostic ultrasound. Moreover, the temperature changes did not appear to be intensity-dependent above a certain intensity threshold, given the trends in the temperature change data at intensities above 8 W/cm^2^.

The temperature change trend for each studied intensity showed minimal variation at intensities from 8 W/cm^2^ to 14 W/cm^2^. The four intensities studied in this range varied in peak temperature change by a range of 0.16 °C, whereas the increase in peak temperature change from 6 W/cm^2^ to 8 W/cm^2^ was substantially larger, at 0.47 °C. Furthermore, there was no significant difference between the mean temperature increases between intensities from 8 W/cm^2^ and 14 W/cm^2^, but there was significance when increasing the intensity from 6 W/cm^2^ to 8 W/cm^2^. While the temperature results at intensities above 8 W/cm^2^ are still below the FDA guideline of 2 °C, we nonetheless recommend that future studies that focus on characterizing the temperature dynamics from intensities of 6 W/cm^2^ to 8 W/cm^2^ be conducted. These findings support that there is no intensity-dependent relationship with skin temperature above a certain intensity threshold, given the lack of a significant difference at intensities above 8 W/cm^2^.

We believe that some of the variation in temperature changes could be due to individual subject variations in surface skin temperatures and the varying efficiencies of the subject’s body when carrying the introduced thermal energy away from the skin. In all cases, the skin temperature increased somewhat during the stimulation period. However, we cannot exclude the possibility that this was partly due to the warming of the transducer itself, or a result of reduced airflow to the tissue in contact with the transducer, which may have slightly hampered ordinary convective skin cooling.

The most important finding, however, was the absence of any reports of unpleasant events or sensations by the subjects during and after the procedure, including any pain or burning sensations. The lack of any subjective findings is further reinforced as the subjects were not able to distinguish between the sham and active transducer. At the end of the experiment, when the thermistor and transducer were disconnected, there were no clinical findings of erythema, swelling, or burns of the skin. These findings reassert the safety of FUS at intensities well above the FDA guideline of I_SPTA.3_ = 720 mW/cm^2^. This serves as evidence of the possibility for future studies utilizing higher FUS intensities, which is critical to gaining a complete understanding of the safety of low-intensity FUS and the possible scope of future uses. Furthermore, while we acknowledge the limitations of the direct application of scapular findings with FUS to tFUS, given the anatomical similarity of the scapula to the temporal window and the lack of a sustained intensity-dependent relationship with increases in skin temperature, these results suggest that skin heating with tFUS at higher intensities does not pose a safety concern. Further studies that begin incrementally increasing tFUS intensities beyond those near the FDA guideline of I_SPTA.3_ = 720 mW/cm^2^ are needed to validate the safety of higher-intensity tFUS.

## 5. Conclusions

FUS at varying intensities above the FDA guideline of I_SPTA.3_ = 720 mW/cm^2^ of living skin and bone does not appear to cause any discomfort during and after stimulation. There was an absence of any skin irritation, including erythema, swelling, or burns, under the transducer after stimulation. Additionally, FUS did not raise the temperature at the skin–transducer interface above 1.5 °C, when varying I_SPTA.3_ up to I_SPTA.3_ = 12 W/cm^2^. This is in line with what would be predicted based on the TIC.

Therefore, FUS at I_SPTA.3_ below these thresholds appears to be safe with regard to the burning and heating of the skin. Further safety studies are recommended to validate the safety of FUS technology at higher intensities.

## Figures and Tables

**Table 1:** Timeline of sonication protocol.

1 min	2 min	1 min	2 min	1 min	2 min	1 min	2 min	1 min
Sonication 1	Break	Sonication 2	Break	Sonication 3	Break	Sonication 4	Break	Sonication 5

**Table 2:** Peak temperature change across each tested FUS intensity level.

Intensity (I_SPTA.3_)	Peak Temperature Change (° Celsius)
6 W/cm^2^	0.95
8 W/cm^2^	1.42
10 W/cm^2^	1.29
12 W/cm^2^	1.45
14 W/cm^2^	1.35

**Table 3:** Marginal mean increase in skin temperature with 95% confidence intervals for each tested FUS intensity.

Intensity (I_SPTA.3_)	Marginal Mean (°C)	95% CI for Mean Difference (°C)	SE (°C)
6 W/cm^2^	0.38	(0.29, 0.47)	0.045
8 W/cm^2^	0.72	(0.63, 0.81)	0.045
10 W/cm^2^	0.63	(0.54, 0.72)	0.045
12 W/cm^2^	0.75	(0.66, 0.84)	0.045
14 W/cm^2^	0.68	(0.59, 0.76)	0.045

## Data Availability

The original contributions presented in this study are included in the article. Further datasets are not readily available because of patient privacy and identification concerns. Further inquiries can be directed to the corresponding author.
